# Insights into the Antimicrobial Mechanism of Piezoelectric Materials

**DOI:** 10.1021/acsomega.5c06348

**Published:** 2025-10-08

**Authors:** Joana Moreira, Žiga Pandur, Margarida Fernandes, Pedro Martins, Vítor Correia, Senentxu Lanceros-Mendez, David Stopar

**Affiliations:** † Physics Centre of Minho and Porto Universities (CF-UM-UP) and LaPMET—Laboratory of Physics for Materials and Emergent Technologies, 56059 University of Minho, Braga 4710-057, Portugal; ‡ Centre of Chemistry, University of Minho, Braga 4710-053, Portugal; § Biotechnical Faculty, University of Ljubljana, Večna pot 111, Ljubljana 1000, Slovenia; ∥ Faculty of Mechanical Engineering, University of Ljubljana, Aškerčeva 6, Ljubljana 1000, Slovenia; ⊥ CMEMS Uminho, LABBELS—Associate Laboratory, University of Minho, Guimarães 4800-058, Portugal; # Centre of Molecular and Environmental Biology, University of Minho, Braga 4710-057, Portugal; ¶ IB-S—Institute for Research and Innovation on Bio-Sustainability, University of Minho, Braga 4710-057, Portugal; ∇ Faculty of Engineering, University of Porto (FEUP), Porto 4200-465, Portugal; ○ BCMaterials, Basque Center for Materials, Applications and Nanostructures, UPV/EHU Science Park, Leioa 48940, Spain; ⧫ Ikerbasque, Basque Foundation for Science, Bilbao 48009, Spain

## Abstract

Physical disruption of bacterial integrity with piezoelectric materials offers a promising alternative to conventional bactericidal chemical treatments. In this study, we investigated the mechanisms of the antibacterial effect of mechanically stimulated poly­(vinylidene fluoride-*co*-trifluoroethylene) (P­(VDF–TrFE)) piezoelectric material on *Escherichia coli* cells with modified cell wall layers. Cells with modified peptidoglycan layer, outer membrane, or extracellular polymer matrix were tested for piezoelectric susceptibility either in direct contact with the piezoelectric material or in suspension after mechanical stimulation of the piezoelectric material, during the exponential and stationary growth phase. The results show that the P­(VDF–TrFE) material can electrostatically inhibit the growth of *E. coli*. The antibacterial efficacy can be further enhanced by the piezoelectric effect under mild mechanical stimulation at 1 Hz. Since most chemical antibacterial agents are effective against exponentially growing bacterial cells, it is a significant finding that piezoelectric stimulation is also very effective against stationary cells. The reduction of surface charges by cell wall modifications increased the resistance of bacteria to the electrostatic effects of P­(VDF–TrFE), but the antibacterial effect could be enhanced by piezoelectricity. Piezoelectric antimicrobial enhancement was most pronounced on cells with disrupted peptidoglycan layer and extracellular matrix removed. Based on the results of this study, one can envision an application of P­(VDF–TrFE)-coated materials on “high-touch” surfaces, such as light switches, doorknobs or countertops, that could be piezoelectrically stimulated by touch, providing an efficient and seamless solution for antibacterial surfaces.

## Graphical abstract

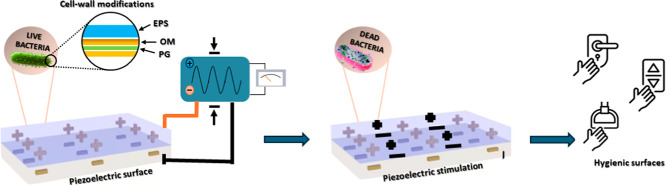

## Introduction

Physical disruption of bacterial cell wall integrity with piezoelectric materials offers a promising alternative to conventional bactericidal, bacteriostatic, and bacteriolytic chemical agents.^1–3^ In contrast to chemical treatments, external physical forces act immediately on bacterial cell wall structures, making the development of bacterial resistance much more difficult.^4,5^ Piezoelectric surfaces offer several advantages, including environmental sustainability, compatibility with various applications such as medical devices, food packaging, and water purification systems, and represent an attractive alternative to conventional antimicrobial strategies.^5–7^

We have previously shown that piezoelectric stimuli can significantly affect the viability of *Escherichia coli*.^8,9^ However, the exact mode of action remains unclear, which limits the optimization and application of piezoelectric stimuli. It is generally recognized that the ability of *E. coli* to resist physicochemical stress in the environment is often closely linked to its multilayered cell wall structure.^10,11^ The cell envelope of *E. coli* consists of several key structures, including the cytoplasmic membrane, the periplasmic space with the peptidoglycan layer, the outer membrane, pili, fimbriae, and flagella, the capsular layer, and the loosely bound extracellular polymeric substances such as the slime layer.^12,13^ Each layer has a unique chemical composition that interacts with the other layers to form a robust, multilayered cell envelope that can withstand a variety of environmental stressors.^13,14^ Among the cell wall components, peptidoglycan is traditionally considered as the primary osmotic pressure bearing element,^15^ and is one of the main targets for antibiotics (i.e., β-lactams) and enzymes (such as lysozyme).^16,17^ The enzymes can alter or destroy the peptidoglycan mesh structure, weakening the cell structure and leading to bacterial lysis.^18^ The charge distribution in the peptidoglycan plays a crucial role in structural stability, defense against antimicrobial agents, and cell signaling.^19^ In addition, the thickness of peptidoglycan layer influences mechanical resistance and susceptibility to osmotic fluctuations. In *E. coli*, for example, the thin peptidoglycan layer (2 to 7 nm) makes the cell more susceptible to physical and mechanical stress.^20^ The outer membrane is another important structural component of the cell wall that, together with the peptidoglycan, helps to resist turgor pressure fluctuations. Its composition is highly asymmetric, with an inner leaflet rich in phospholipids and an outer leaflet composed predominantly of polyanionic lipopolysaccharides (LPS), which provide a negative surface charge.^21,22^ *E. coli* produces other extracellular polymers, including capsular polysaccharides (CPS), exopolysaccharides (EPS), and extracellular DNA (eDNA), all of which are predominantly negatively charged. These polymers form an electrostatically cohesive matrix that improves cell adhesion and provides protection against environmental threats.^23^ The effect of piezoelectricity on bacterial cells was previously studied by Brito-Pereira et al., who observed *E. coli* cells with irregular morphology after applying a mechanical stimulus of 1 Hz.^24^ How the different cell wall components are influenced by piezoelectric effects is not known.

Recently, piezoelectric polymers such as poly­(vinylidene fluoride-*co*-trifluoroethylene) (P­(VDF–TrFE)) have been used as antimicrobial surfaces based on their ability to generate electrical potential fluctuations in response to mechanical stimulation.^25–27^ When the material is deformed, it can induce microvibrations that exert mechanical stress on bacterial cells, which can lead to membrane disruption or detachment of the cells from surface.^26,28^ The antibacterial mechanisms of piezoelectric materials in general involve three primary pathways such as generation of reactive oxygen species (ROS) under piezoelectric conditions; direct bacterial inactivation through electrical stimulation; or interaction between surface charges of piezoelectric materials and different bacterial cell wall structurers leading to cell death.^29^ In this work we will specifically address the interaction between the surface charges of piezoelectric material and bacterial cell wall surface structures.

The *E. coli* was exposed to a mechanically stimulated piezoelectric P­(VDF–TrFE) material. To determine which bacterial cell wall component is most sensitive to piezoelectric stimuli, we selectively modified different cell wall layers and molecular components (EPS, outer membrane and peptidoglycan layer). The integrity of the intact cells and the cells with modified cell envelopes in direct contact with the piezoelectric P­(VDF–TrFE) material or the cells suspended in the medium, was tested after application of mechanical stimuli, during the exponential and stationary growth phases. This study represents a significant advance by demonstrating that the copolymer P­(VDF–TrFE), in addition to its widely recognized piezoelectric properties, also exhibits good antimicrobial activity. By developing electroactive surfaces from this piezoelectric material, stimulated through physical methods and without the use of chemical agents, it is possible to confer a multifunctional character to this material. This approach positions P­(VDF–TrFE) as a promising alternative for smart surface coatings, particularly in biomedical applications, where the aim is to combine functional performance with antimicrobial protection.

## Materials and Methods

### Materials

Poly­(vinylidene fluoride-*co*-trifluoroethylene), P­(VDF–TrFE), 70/30 was purchased from PiezoTech (Lyon, France). *N*,*N*-dimethylformamide (DMF, pure grade) was used as solvent for P­(VDF–TrFE) and purchased from Merck.

### Samples Preparation and Characterization

A solution containing 15 wt % P­(VDF–TrFE) powder was prepared by dissolving the polymer in DMF under magnetic stirring. To accelerate dissolution, the mixture was heated to 30 °C for the first 30 min. The solution was then stirred continuously until it became uniform and transparent. The homogeneous P­(VDF–TrFE) solution was then applied evenly to clean glass substrates using the doctor blade method with a ≈450 μm spacer. The coated samples were placed in an oven (JP Selecta, model 2000208, Barcelona, Spain) at 210 °C for 10 min to ensure complete evaporation of the solvent. The films were then removed from the oven and cooled to room temperature. To optimize the macroscopic piezoelectric response, the films were corona poled by placing the sample at a distance of about 2 cm from the corona grid and exposing it to a voltage difference of 15 kV at 120 °C for 1 h. The samples were then cooled to room temperature while maintaining the applied potential. The prepared film is shown in Figure S1. As described in the literature, the prepared electroactive P­(VDF–TrFE) exhibits hydrophobic^24^ properties and has a degree of crystallinity (Χ_c_) of ≈28%.^29^ In addition, as described in the literature, the P­(VDF–TrFE) films, show typical absorption bands, including a strong peak at 840 cm^–1^, a band at 510 cm^–1^, and another at 1279 cm^–1^, all of which are exclusively associated with the electroactive β-phase of the polymer.^25^ The formation of the β-phase in P­(VDF–TrFE) is strongly dependent on the synthesis and processing conditions. The VDF/TrFE ratio, solution processing, and thermal annealing promote the all-trans conformation of the polymer chains, favoring the stabilization of the polar β-phase over the nonpolar α-phase. Additionally, factors such as cooling rate and film deposition method can influence the orientation and crystallinity of the β-phase, contributing to the lamellar microstructure observed in cross-sectional SEM images. The main characteristics of the electroactive polymer used for the antimicrobial assays are listed in Table 1.

**Table 1:** Relevant Characteristics of the P­(VDF–TrFE) Film Used in the Antimicrobial Assays

polymer	morphology^30^	contact angle (°)^24^	Χ_c_ (%)^29^	FTIR bands wavenumber (cm^–1^)^25^	piezoelectric coefficient *d*_33_ (pC N^–1^)^31^
poly(vinylidene fluoride-*co*-trifluoroethylene) (P(VDF–TrFE))	smooth and compact	≈90°	≈28%	β-phase:	–38
				510 cm^–1^	
				840 cm^–1^	
				1279 cm^–1^	

### Growth and Preparation of Bacteria with Weakened Cell Wall Layers

Unless otherwise stated, *E. coli* MG1655 (*wt*) was used for the experiments. The strain contains an IPTG (isopropyl β-d-1-thiogalactopyranoside)-inducible green fluorescent protein (GFP) expression system. Frozen stock cultures stored at −80 °C were plated on lysogeny broth (LB) agar plates supplemented with kanamycin (Kn) (50 μg/mL). The bacterial colonies from the LB plates were used to inoculate overnight cultures, which were incubated at 37 °C with shaking at 200 rpm under aerobic conditions. The overnight culture was used as a cell culture in the stationary growth phase. For the exponential growth phase, the 1% inoculum of the overnight culture was transferred to fresh LB medium containing Kn (50 μg/mL) and incubated at 37 °C with shaking at 200 rpm until the culture reached the exponential growth phase (OD_600_ of approximately 0.5 a.u.). After obtaining the exponential or stationary cells, a series of modifications were made to the cell wall layers, as described below.

### Modification of the Composition of the Extracellular Layer

For extracellular layer modification, we used an isogenic mutant of *E. coli* MG1655 strain (*eps*-), which lacks the ability to produce β-1,6-*N*-acetyl-d-glucosamine (PGA), curli proteins, and colanic acid (Table 2).

**Table 2:** Bacterial Strains Used in the Experiments^a^

strains	name	genotype	phenotype	extracellular polysaccharides produced by the strain
E. coli MG1655	*wt*	*wt*	Wild type	PGA, curli
E. coli MG1655	*eps*-	ΔcsgA	deletion of csgA, pgaC and lon genes, insertion of kanamycin resistance gene, cpsE gene interrupted by tetracycline resistance gene	does not produce PGA, curli, colanic acid
		ΔpgaC		
		Δlon::kan cpsE::Tn10		

a Adapted from ref 32. Copyright 2019 Royal Society of Chemistry.

### Modification of the Outer Membrane Layer

For modification of the outer membrane, EDTA was used to sequester divalent Ca^2+^ and Mg^2+^ ions. Chelation of divalent cations with EDTA removes divalent metal ions such as Ca^2+^ and Mg^2+^ from their binding sites in the outer membrane, thereby weakening LPS interactions.^33^ Cells in the exponential or stationary growth phase were harvested by centrifugation at 5000 rpm for 5 min. The resulting pellet was resuspended in 0.8 M sucrose. EDTA was then added to the cell suspension at a final concentration of 3 mM, together with 25 mM Tris buffer (pH 8.0).

### Modification of the Peptidoglycan Layer

The antibiotic cephalexin was used to modify the peptidoglycan layer. The cephalexin targets the penicillin-binding protein (PBP3, FtsI), which is involved in the transpeptidase cross-linking of peptide chains necessary for the strength and rigidity of the peptidoglycan.^34,35^ Cephalexin was added to the exponential and stationary cells at a final concentration of 50 μg/mL, and the suspension was incubated for 30 min at 37 °C with shaking at 200 rpm. Alternatively, the peptidoglycan was modified by lysozyme, an enzyme that cleaves the glycosidic bonds in the peptidoglycan. Lysozyme was added to the cell culture in the exponential or stationary growth phase at a final concentration of 100 μg/mL. The cell suspension was then incubated for 30 min at 37 °C with shaking at 200 rpm. All cell samples (with the exception of the outer membrane modification) were harvested by centrifugation at 5000 rpm for 5 min prior to further experiments. The resulting pellet was resuspended in 0.9% saline solution.

### Antimicrobial Activity of P­(VDF–TrFE) under Electrostatic and Piezoelectric Conditions

To evaluate the antimicrobial activity of P­(VDF–TrFE) films, the material was first cut into circles with a diameter of 13 mm, washed with distilled water and dried. A total volume of 500 μL of each bacterial suspension was added to a 24-well plates containing the P­(VDF–TrFE) material. Wells without the material were used as controls for bacterial growth. Under electrostatic conditions (based on the P­(VDF–TrFE) surface charges of the poled materials), the well plates were incubated at 37 °C for 2 h without agitation or external stimuli. The electrostatic conditions refer to permanent charge on the P­(VDF–TrFE) films. Under piezoelectric conditions, the 24-well plates were placed on a laboratory made bioreactor system^30^ with mechanical stimulation for a period of 2 h at 37 °C. The mechanical bioreactor used in this assay was designed and built in our laboratory. It applies continuous vertical vibrations and oscillatory movements, and is equipped with three support platforms, a vibratory actuator, and a frequency control adjustable between 1 and 10 Hz. These frequencies were selected based on the literature for enhanced antimicrobial effect.^8,24,30,36^ The bioreactor has the capacity to accommodate up to three 24 or 48 well plates.

### Evaluation of the Viability of Bacterial Cells in Suspension

To determine the number of surviving bacteria after 2 h, the resulting suspension was serially diluted in a sterile buffer solution. Then, 10 μL of each dilution of the corresponding samples were added to an LB agar plate supplemented with Kn (50 μg/mL) and incubated for 24 h at 37 °C. Antimicrobial activity was determined for each condition and compared to control cells incubated without any mechanical stimuli or P­(VDF–TrFE) material. The results were expressed as a percentage reduction in the number of viable bacterial cells compared to the control.

### Bacterial Viability on the P­(VDF–TrFE) Surfaces

To assess the viability of the bacterial cells attached to the P­(VDF–TrFE) surface, the samples were examined using a Zeiss Axio Observer 7 (Zeiss, Jena, Germany) fluorescence microscope with LED illumination system (X-Cite XYLIS, Pittsburgh, United States). IPTG was added to the wild-type strain, at a final concentration of 40 μM to induce the synthesis of gfp. The *eps*- mutant was stained with the fluorescent dye SYTO-9 to fluorescently label the cells. In both cases, a 480 nm excitation light source and a 540–580 nm emission filter were used. To identify dead cells, the samples were additionally stained with propidium iodide (PI), using a 560 nm excitation light source and a 593–668 nm emission filter. The sample was prepared by placing the P­(VDF–TrFE) foil on a microscope slide and covering it with a coverslip. To prevent the sample from drying out, the slides were sealed with VALAP sealant, a mixture of Vaseline, lanolin, and paraffin wax. After 15 min of incubation at room temperature, the samples were examined under a microscope. Microscopic images were captured from six random locations on each slide for each treatment time using a 40× magnification lens. The captured images were then analyzed using ImageJ 1.53c software, and live and dead bacterial cells were counted.

### Contact Angle Measurements

The wettability of the materials for different bacterial suspensions (overnight and exponential bacteria) for the wild-type and cells with modified cell wall structures was evaluated by measuring the contact angle at room temperature. Contact angles were measured using the Krüss DSA25E (Krüss Optronic GmbH, Hamburg, Germany) drop shape analyzer system. For each sample, the contact angle was determined five times at different locations, and the results were presented as the average values together with the corresponding standard deviations.

### Zeta Potential Measurements

Zeta potential measurements for overnight and exponential bacterial suspensions of wild-type and *E. coli* cells with modified cell wall structures were performed using the Zetasizer NANO ZS-ZEN3600 (Malvern Instruments Limited, Malvern, UK). Cell samples were freeze-dried in saline or sucrose solution. Prior to measurements, samples were resuspended in ultrapure water and each sample was measured five times at pH = 6. The manufacturer’s software (Zetasizer 7.13) was used to estimate the zeta potential values.

### Statistical Analysis

The results are presented as the average of the individual measurements with the respective standard deviations and analyzed using GraphPad Prism version 9.0.0 for Windows (Graph Pad Software, San Diego, United States). Statistical significance was determined using a one-way analysis of variance (ANOVA), followed by an unpaired, two-tailed Student’s *t*-test.

## Results

The surface and cross-section morphology of the P­(VDF–TrFE) samples used in this study are shown in Figure 1. When the polymer crystallized from the melt after evaporation of the solvent at 210 °C, a compact film with a smooth surface was obtained.^24,30^ The crystallization of P­(VDF–TrFE) in the selected copolymer ratio of 70:30 (70% vinylidene fluoride (VDF) and 30% trifluoroethylene (TrFE)) always leads to the ferroelectric β-crystalline phase. This is due to the presence of fluoride ions in the TrFE monomers, which cause a steric hindrance in the polymer structure and favor an all-trans polymer chain conformation.^25^ The lamellar pattern observed in the cross-sectional SEM image is attributed to the semicrystalline nature of P­(VDF–TrFE), where crystalline lamellae are embedded in an amorphous matrix, formed during solution processing and thermal annealing. The application of mechanical stimuli to P­(VDF–TrFE) films induces dipolar changes leading to a piezoelectric response.^37^ In this study, a mechanical stimulus using a mechanical bioreactor vibrating vertically at a frequency of 1 Hz was applied to induce piezoelectricity in P­(VDF–TrFE) films in the presence of bacterial cultures.^24,30^

**Figure 1:**
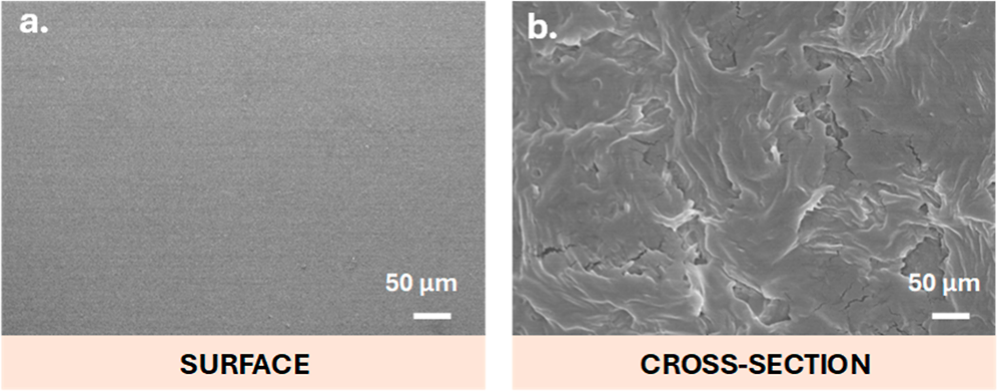
Representative surface (a) and cross-section (b) SEM images of the P­(VDF–TrFE) film obtained after evaporation of the solvent at 210 °C and cooling down to room temperature.

Piezoelectric stimulation enhanced the antibacterial activity of P­(VDF–TrFE), as shown in Figure 2a. The samples were exposed to the mechanical stimuli for 2 h (piezoelectric conditions), while the control samples were not treated mechanically (electrostatic conditions). Microscopic analysis showed that the fraction of dead cells on the surface of the piezoelectric material increased significantly in the presence of mechanical vibrations for both exponential and stationary bacteria. Nevertheless, the piezoelectric effect was greater for the exponential cells attached to the P­(VDF–TrFE) surface.

**Figure 2:**
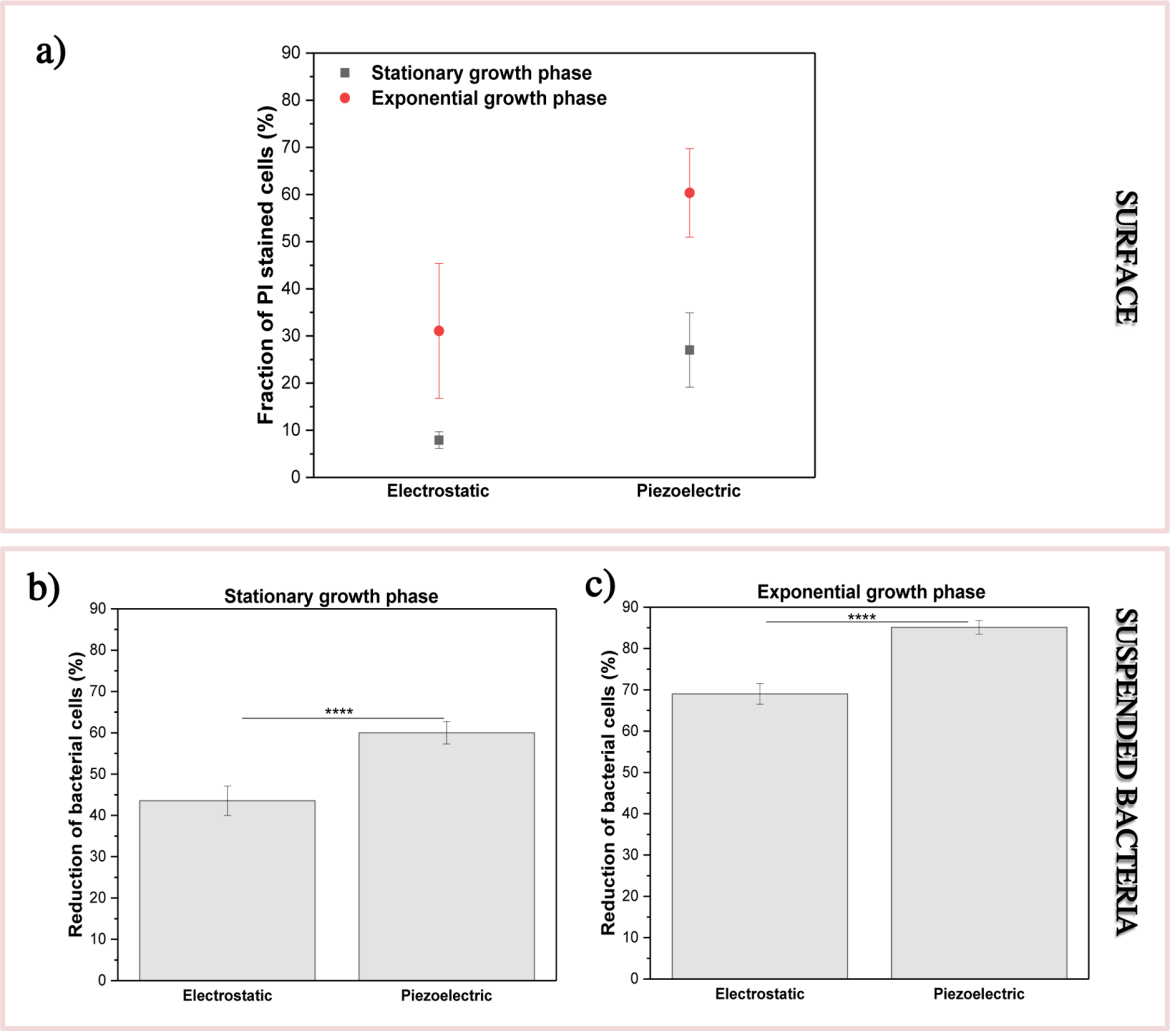
P­(VDF–TrFE) antimicrobial activity against wild-type *E. coli* (*wt*): (a) stationary and exponential bacteria attached to P­(VDF–TrFE) under electrostatic and piezoelectric (1 Hz) conditions; (b,c) the effect of P­(VDF–TrFE) on suspended stationary (b) and exponential (c) bacteria under electrostatic and piezoelectric conditions. The results represent five individual measurements. ****P < 0.0001.

The electrostatics and piezoelectricity of P­(VDF–TrFE) also affected the suspended bacteria (Figure 2b,c). Under the electrostatic conditions, P­(VDF–TrFE) inhibited the growth of the suspended bacteria, especially in the exponential cells. The electrostatic antibacterial effect was significantly enhanced by the piezoelectricity. To evaluate the mechanism behind the electrostatic and piezoelectric effect on bacteria, we tested *E. coli* cells with different modifications of the cell wall layers. The effect was evaluated for bacteria with reduced abundance of major capsular polymers (*eps*- mutant), a damaged outer membrane (treated with EDTA), and an impaired peptidoglycan layer (treated with the antibiotic cephalexin or lysozyme). The results of the contact angle measurement (Figure 3) show that a significant increase in hydrophobicity was observed in the stationary cells when treated with EDTA. In the exponential cells, the absence of EPS in the *eps*- mutant increased the hydrophobicity. The EPS is largely composed of hydrophilic residues and its removal should increase hydrophobicity.^38^ On the other hand, treatment with lysozyme (Lys) increased the hydrophilicity of the cell surface compared to exponentially grown wild-type cells.

**Figure 3:**
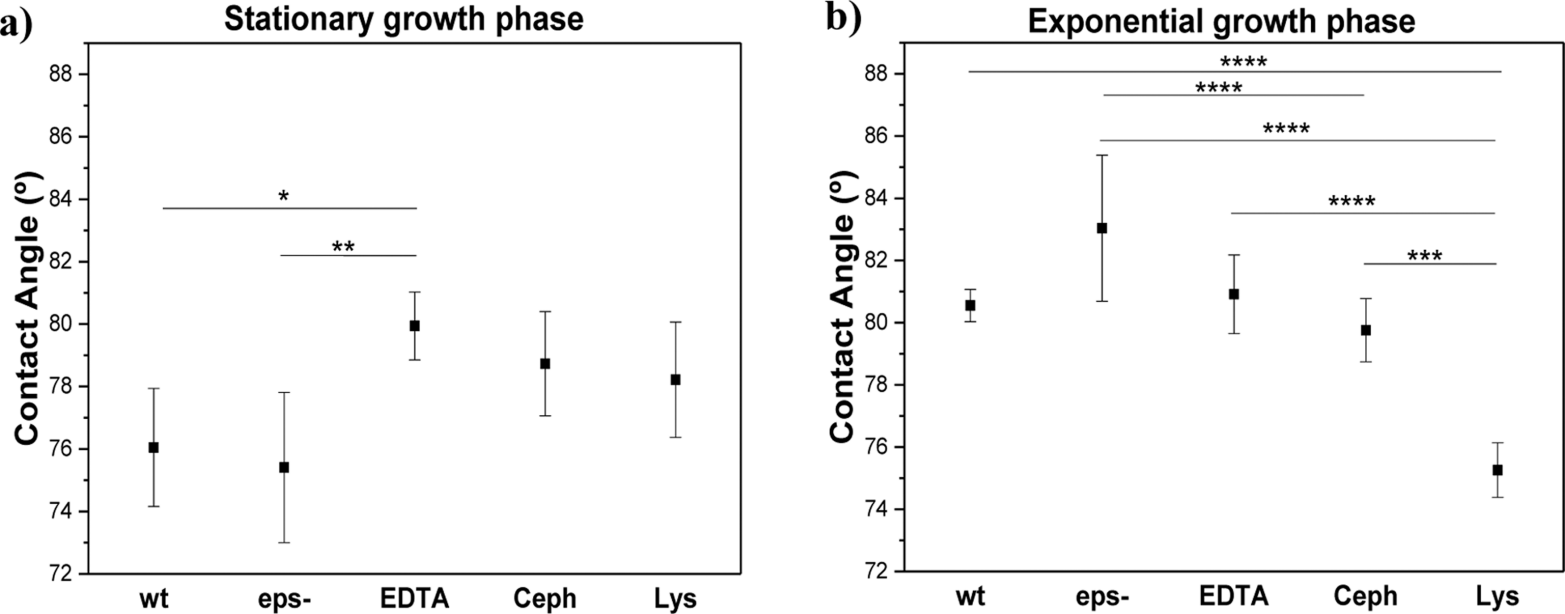
Contact angle measurement for the wild-type *E. coli* strain (*wt*), cells with removed EPS (*eps-*), modified outer membrane (EDTA treatment), and modified peptidoglycan layer (Cephalexin (Ceph) or lysozyme (Lys) treatment) for (a) stationary and (b) exponential growth phase. The results represent five individual measurements. *P < 0.1, **P < 0.01, ***P < 0.001, ****P < 0.0001.

The results of the zeta potential measurement (Figure 4) show a large effect of the cell wall modifications on the electrostatic properties of the cells. Removal of EPS increased the zeta potential, and the effect was significant in the stationary cells. Accordingly, removal of peptidoglycan by treatment with lysozyme led to a large increase in zeta potential in both exponential and stationary cells. In contrast, treatment with EDTA, which disrupts the outer *E. coli* membrane, significantly decreased zeta potential in the exponential cells, but had a much smaller effect on the stationary growth cells. In the presence of EDTA, the outer membrane loses its structural integrity and large amounts, up to 50% of the total LPS, are released into the solution.^39–41^ In contrast, treatment with cephalexin had only a limited effect on cell surface charge.

**Figure 4:**
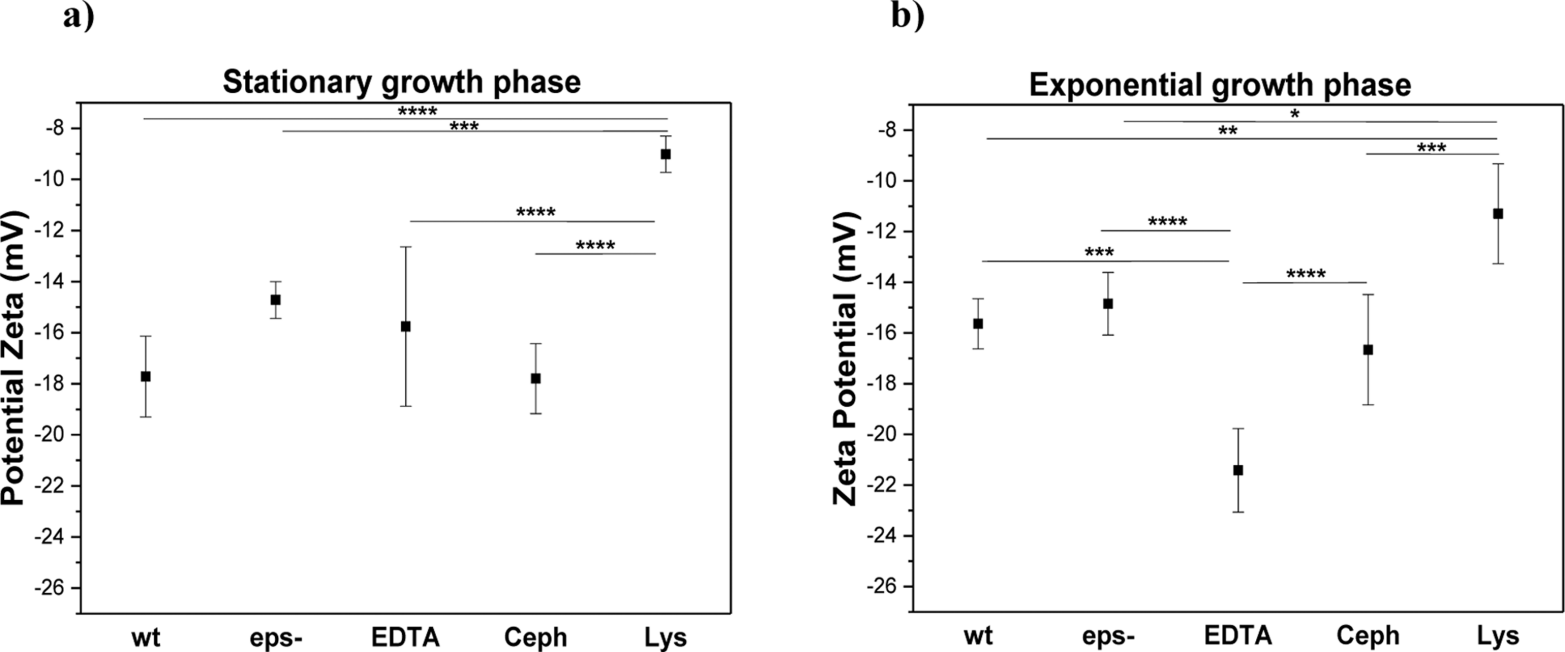
Zeta potential measurements for the wild-type *E. coli* strain (*wt*), cells with removed EPS (*eps-*), modified outer membrane (EDTA treatment), and modified peptidoglycan layer (cephalexin (Ceph) or lysozyme (Lys) treatment) for (a) stationary and (b) exponential cells. Results represent five individual measurements. *P < 0.1, **P < 0.01, ***P < 0.001, ****P < 0.0001.

Changes in the surface properties of cells with modified cell walls influenced bacterial susceptibility to electrostatic and piezoelectric effects. The susceptibility of cells with compromised cell wall layers to the electrostatic and piezoelectric stimuli is shown in Figure 5a. The results indicate that *E. coli* cells with removed cell wall layers survive better in the presence of P­(VDF–TrFE) than the wild-type cells. With the induction of piezoelectricity in P­(VDF–TrFE) by dynamic mechanical stimulation, the antimicrobial effect increased in all samples. When we compared the piezoelectric effect with the electrostatic antibacterial effect, the piezoelectric enhancement was greater in cells with modified cell wall layers than in the wild-type (Figure 5b). This indicates that piezo-electrodynamic forces have a major impact on bacterial viability when cell wall structures are weakened or removed. The largest piezoelectric enhancement was measured in the absence of the extracellular polymer matrix (Figure 5c,d). Qualitatively similar piezoelectric enhancement response was observed for the exponential and stationary cells. The notable difference was a weaker piezoelectric enhancement for peptidoglycan treated bacteria in the exponential growth phase.

**Figure 5:**
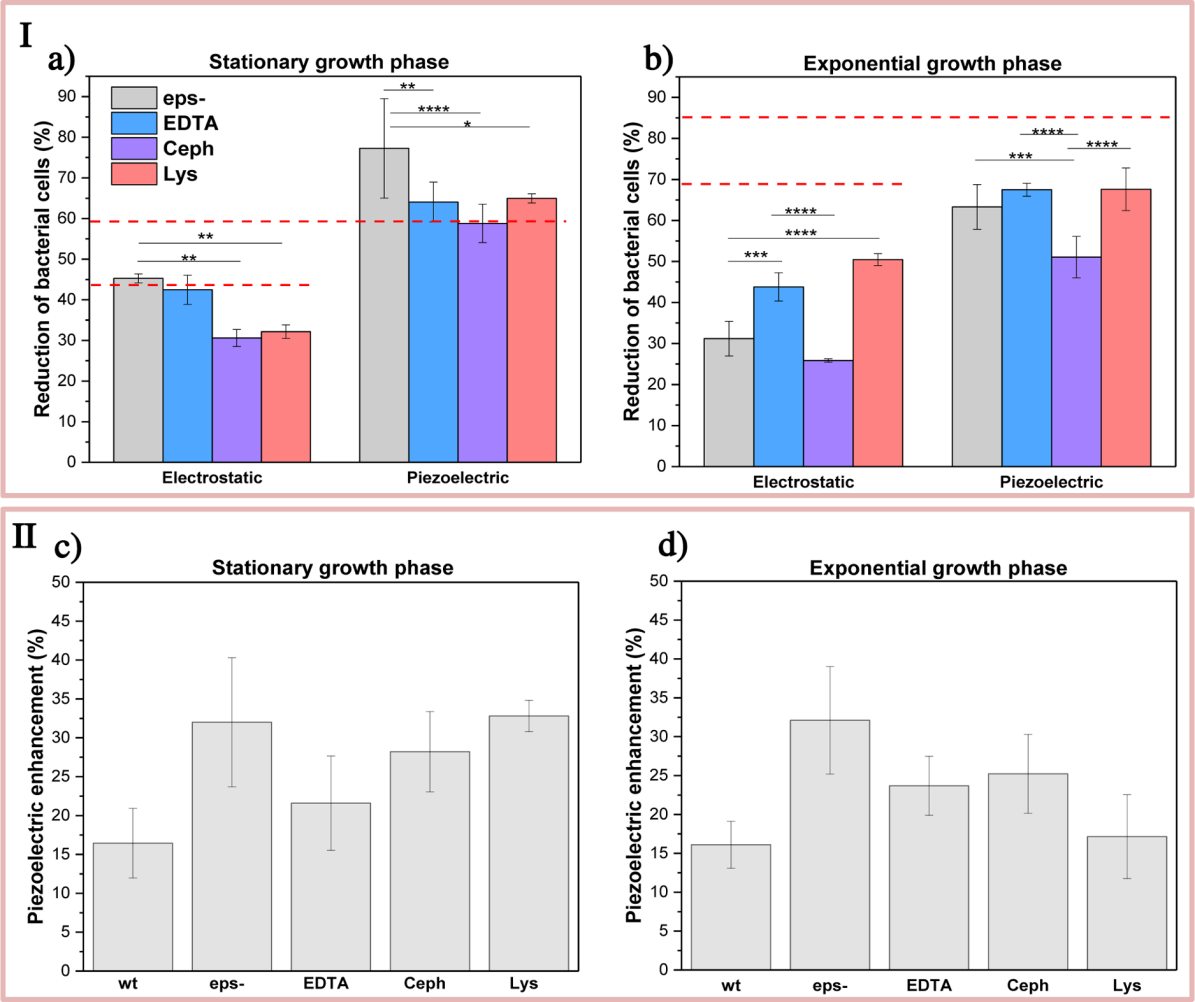
(I) Antimicrobial activity against *wt* *E. coli*, cells with removed EPS (*eps-*), modified outer membrane (EDTA treatment), and modified peptidoglycan layer (cephalexin (Ceph) or lysozyme (Lys) treatment), measured as reduction of fraction of viable bacterial cells (%) relative to the control for (a) stationary and (b) exponential bacteria under electrostatic and piezoelectric conditions. The results represent five independent measurements. **P < 0.01; ***P < 0.005; ****P < 0.0001. The red dashed line represents the reduction of bacterial cells for wild-type *E. coli* (*wt*) under electrostatic and piezoelectric conditions for the stationary and exponential cells. (II) Piezoelectric antibacterial enhancement (%) relative to the electrostatic effect on suspended bacteria after 2 h of mechanical stimulation for the stationary (c), and exponential bacteria (d).

## Discussion

In this study, we investigated how the antimicrobial properties of P­(VDF–TrFE) surfaces can be enhanced by mechanical stimulation that induces piezoelectricity. Our results show that both electrostatic and piezoelectric forces contribute to bacterial inactivation. In particular, we found that piezoelectric stimulation can significantly enhance the antimicrobial electrostatic efficacy of P­(VDF–TrFE), especially in bacterial cells with modified envelope structures.

Piezoelectric activation induces local surface charge fluctuations and mechanical stress, that compromises the integrity of the bacterial cell membrane,^30^ as shown by the increased uptake of propidium iodide (a positively charged dye impermeable to intact membranes) under piezoelectric conditions.^42^ Piezoelectricity generates microvibrations and varying electrical surface charges that exert mechanical and electrical stress on bacterial cells, potentially leading to cell death and detachment from the surface.^6^ While piezoelectricity decreased the number of viable cells on the P­(VDF–TrFE) surface, the bacterial surface coverage results showed no significant difference between the electrostatic and piezoelectric conditions (Figure S2), suggesting that the piezoelectric stimulation conditions are not strong enough to detach bacterial cells.

An important finding of this study is that electrostatics and piezoelectricity also affect suspended bacteria. This effect can be attributed to the positive charges on the surface of P­(VDF–TrFE), which likely interfere with the negative charges on the bacterial surface, as has been shown with other charged cationic compounds that effectively inhibit bacterial growth by disrupting the cell membrane.^43,44^ The surfaces of *E. coli* are negatively charged due to the dissociation of carboxyl and phosphate groups in the peptidoglycan and lipopolysaccharides.^45,46^ Since electrostatics and piezoelectricity affect both attached and suspended bacteria, piezoelectric materials are expected to interact with charged bacterial surface components via long-range electrostatic forces that reduce the integrity of the cell membrane and allow the impermeable propidium iodide to pass through the cell membrane. The piezoelectric effect was larger in the exponential cells. It is known that during the exponential phase, the active division of *E. coli* can be easily impaired, leading to cell death.^47–49^ The results suggest that active division could also be impaired by the piezoelectric effect. When the material is mechanically stimulated, it generates an electric potential due to its intrinsic piezoelectric properties. This localized electric field can compromise bacterial cell membranes and interfere with essential metabolic processes, ultimately leading to bacterial inactivation. Therefore, it is hypothesized that improving the piezoelectric performance of the composite would further enhance its antimicrobial efficacy. Previous studies have shown that the incorporation of nanofillers, such as nanoparticles or carbon-based materials, can significantly improve the piezoelectric performance of PVDF and its copolymers. By promoting crystallinity, facilitating dipole alignment, or increasing local stiffness, these nanofillers improve the electrical potential generated upon mechanical stimulation. Notably, studies have demonstrated that incorporating optimized nanofillers into P­(VDF–TrFE) can dramatically boost piezoelectric output. Park and Yaseen^50^ reported that 4 wt % PZT produced a β-phase of ∼97%, yielding a *d*_33_ ≈ 78 pC/N. Similarly, WS_2_ nanosheets at 2 wt % elevated β-phase from ∼64% to ∼86%.^51^ As a result, higher piezoelectric performance is expected to lead to greater membrane disruption and have a more pronounced antibacterial effect.^51–53^

Since the effect of the P­(VDF–TrFE) surface on bacterial cells depends on the electrostatic interaction, we have modified or removed selected electrostatic cell wall layers of *E. coli* cells. This should in principle reduce the negative surface charge of the bacteria and consequently decrease the susceptibility to electrostatic effects, as the electrostatic coupling between P­(VDF–TrFE) and bacteria is lower. The results of the lower electrostatic antibacterial effect on cell wall modified cells indeed show a lower electrostatic coupling between P­(VDF–TrFE) and modified bacteria. Since *E. coli* cannot afford to lose its cell wall without significantly compromising its fitness, this is a disadvantage that makes *E. coli* wild-type inherently susceptible to the electrostatic and piezoelectric effects of P­(VDF–TrFE).

The main finding of this study is that piezoelectricity enhances the electrostatic antibacterial effect of P­(VDF–TrFE). Mechanical stimulation induce a piezoelectric response that enhances antibacterial activity for both surface-attached and suspended cells. Importantly, the enhancement was greater for bacteria with cell wall modification, although the absolute efficacy of piezoelectricity was lower than for wild-type cells. This suggests that piezo-electrodynamic forces play a dominant role when cell envelope structures are weakened or absent. The electrodynamic interactions can potentially lead to the removal of membrane lipids, resulting in distortion of cell shape, physical damage to the cell membrane and ultimately cell death.^52^ The strongest piezoelectric enhancement in cells in stationary phase was observed in EPS-deficient and lysozyme-treated cells. This correlates with the largest structural rearrangements and the highest increase in zeta potential observed. The extracellular polymer matrix plays an important role in long-term protection (i.e., in the stationary cells) but much less in rapid cell division (in the exponential cells).^53,54^ Under the conditions of piezoelectric stimulation, the absence of extracellular polysaccharides in the stationary growth phase leaves the bacteria unprotected and more susceptible to the external mechano-electrical stimuli that trigger the cell death mechanism. These results suggest that the absence of protective compounds in *E. coli* bacterial cells, such as PGA, curli, and colanic acid, makes the cells more susceptible to piezoelectricity. On the other hand, the lysozyme-weakened peptidoglycan layer is more susceptible to electrical and mechanical perturbations, resulting in increased membrane permeability, as evidenced by increased propidium iodide uptake and increased antimicrobial efficacy.^55^

Although our study primarily emphasizes the interaction between surface charges of piezoelectric materials and bacterial cell wall structures, it is important to note that other mechanisms may also contribute to antibacterial activity. The generation of reactive oxygen species (ROS) under piezoelectric conditions has been widely reported as a potent antibacterial pathway, since ROS can oxidize cellular components and disrupt vital metabolic processes.^26,56^ When piezoelectric materials are mechanically deformed (e.g., by compression, vibration, or ultrasonic stimulation), they generate surface polarization charges due to the piezoelectric effect. These charges can result in redox reactions with surrounding molecules, particularly oxygen and water, leading to the formation of ROS.^57^ In addition, direct electrical stimulation arising from piezoelectric activity may induce membrane depolarization or leakage, further compromising bacterial viability.^57–59^

These results obtained in this study are in line with the growing interest in electroactive biomaterials for biointerface applications, especially in contexts where passive antibacterial strategies are insufficient. The interaction between the electrically active polymer surface and the bacterial envelope structures, particularly the cell wall-compromised structures, provides new insight into how mechano-electrical stimulation disrupts microbial viability which is important for effective antibacterial surface design. Based on the results of this study, one can envision applications of P­(VDF–TrFE) coated materials on “high-touch” surfaces (e.g., light switches, door handles, and countertops) that could be piezoelectrically stimulated by touch, providing an efficient and seamless solution for antibacterial surfaces.

## Conclusions

The electroactive P­(VDF–TrFE) materials show a good electrostatic antibacterial effect on *E. coli* cells. The electrostatic antibacterial effect can be reduced by selective removal of cell wall layers. However, the reduced antibacterial electrostatic efficacy of P­(VDF–TrFE) can be significantly enhanced by mild mechanical stimulation at a frequency of 1 Hz, which induces mechano-electrical surface changes based on the piezoelectric properties of the material. The relative enhancement of the piezoelectric antibacterial effect over the electrostatic effect depended on the cell wall composition and was most pronounced when peptidoglycan and EPS cell wall layers were removed. Both the electrostatic and piezoelectric effects were dependent on the physiological state of the bacterial cells. Importantly, the results show that the piezoelectric stimulus is very effective against the stationary cells.

## Supplementary Material



## Notes

Digital camera photograph of the synthesized P­(VDF–TrFE) film (Figure S1) and results of cell coverage of bacteria cell *E. coli* on P­(VDF–TrFE) film (Figure S2) (PDF)

## Abbreviations

*E. coli*: *Escherichia coli*
P­(VDF–TrFE): poly­(vinylidene fluoride-*co*-trifluoroethylene)
LPS: lipopolysaccharides
CPS: capsular polysaccharides
EPS: exopolysaccharides
eDNA: extracellular DNA
EDTA: ethylenediaminetetraacetic acid
DMF: *N*,*N*-dimethylformamide
IPTG: isopropyl β-d-1-thiogalactopyranoside
