# Supplementary material for: Insights into the Antimicrobial Mechanism of Piezoelectric Materials
Source: ACS Omega. 2025 Oct 8;10(41):48642–51. doi: 10.1021/acsomega.5c06348 (PMC12547748; doi:10.1021/acsomega.5c06348)
Supplement: Supplementary file 1 [file ao5c06348_si_001.pdf]

Different resistance of bacterial surface structures to electrical stimulation from piezoelectric materials

Joana Moreira[a,b], Žiga Pandur[d], Margarida Fernandes[e], Pedro Martins[f,g], Vítor Correia[h] , Senentxu Lanceros-Mendez[a,i,j], David Stopar[c*]*

[a]Physics Centre of Minho and Porto Universities (CF-UM-UP) and LaPMET - Laboratory of Physics for Materials and Emergent Technologies, University of Minho, Braga 4710-057, Portugal

[b]Centre of Chemistry, University of Minho, 4710-053 Braga, Portugal

[c]University of Ljubljana, Biotechnical Faculty, Večna pot 111, 1000 Ljubljana, SI-Slovenia

[d]University of Ljubljana, Faculty of Mechanical Engineering, Aškerčeva 6, 1000 Ljubljana, SI Slovenia

[e]CMEMS Uminho, University of Minho, Guimarães 4800-058, Portugal; LABBELS – Associate Laboratory, Guimarães 4800-058, Portugal

[f]Centre of Molecular and Environmental Biology, University of Minho, Braga 4710-057, Portugal

[g]IB-S – Institute for Research and Innovation on Bio-Sustainability, University of Minho, Braga 4710-057, Portugal

[h]Faculty of Engineering, University of Porto (FEUP), 4200-465 Porto, Portugal

[i]BCMaterials, Basque Center Centre for Materials, Applications and Nanostructures, UPV/EHU Science Park, Leioa 48940, Spain

[j]Ikerbasque, Basque Foundation for Science, 48009 Bilbao, Spain

*Corresponding authors

SUPPLEMENTARY INFORMATION

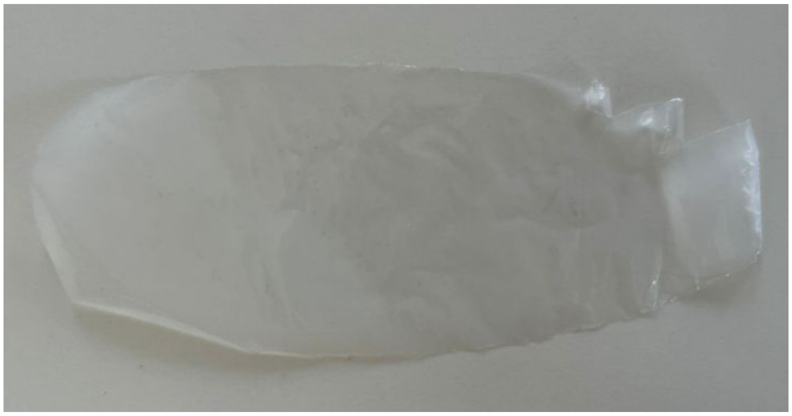

Supplementary Figure 1. Digital camera photograph of the synthesized P(VDF-TrFE) film.

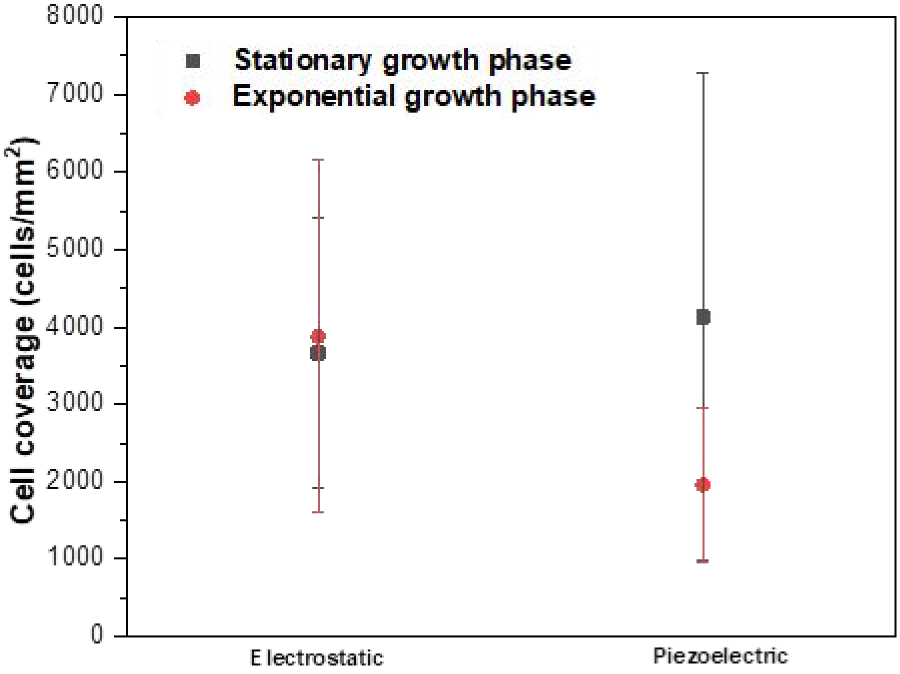

Supplementary Figure 2. Cell coverage of bacteria cell *E. coli* on P(VDF-TrFE) film after 2h electrostatic and piezoelectric conditions.
